# Supplementary material for: Identification and Allelopathy of Green Garlic (Allium sativum L.) Volatiles on Scavenging of Cucumber (Cucumis sativus L.) Reactive Oxygen Species
Source: Molecules. 2019 Sep 7;24(18):3263. doi: 10.3390/molecules24183263 (PMC6767350; doi:10.3390/molecules24183263)
Supplement: Supplementary file 1 [file molecules-24-03263-s001.zip › Supporting Information-Fan Yang/Table S1.pdf]

Table S1. Total 17 compounds of green garlic volatile compounds of cutting segments (in sealed desiccator)

Compound Name	**RT** (min)	**Area%**	Molecular Formula	Molecular Weight
Hexamethyl cyclotrisiloxane	5.18	0.43	C6H18O3Si3	222
Diallyl sulfide	6.36	0.35	C6H10S	114
Methyl propenyl disulfide	7.87	1.53	C4H8S2	120
2,6-Dimethyl-undecane	8.30	0.46	C13H28	184
3-Ethyl-2-methyl-heptane	8.46	1.77	C10H22	142
Octamethyl cyclotetrasiloxane	9.69	0.45	C9H28O3Si4	296
6-Methyl-octadecane	10.16	0.67	C10H23NO	173
4-Methyl-decane	10.73	1.17	C11H24	156
1-Ethyl-2,2,6-trimethyl cyclohexane	11.33	0.44	C11H22	154
2-Hexyl-1-octanol	12.80	0.75	C14H30O	214
2-Methyl-trans-decalin	13.19	0.47	C11H20	152
p-Menthane-1,2,3-triol	13.22	0.33	C10H20O3	188
6-Methyl-octadecane	13.47	0.34	C23H44O3	368
1,1-bis(dodecyloxy)-Hexadecane	14.87	1.57	C40H82O2	594
2,6-Dimethyl-undecane	15.64	0.97	C13H28	184
2-Butyl-1,1,3-trimethyl-cyclohexane	15.84	1.32	C13H26	182
3,7,11-Trimethyl-1-dodecanol	17.16	0.64	C15H32O	228
