# Supplementary material for: Identification and Allelopathy of Green Garlic (Allium sativum L.) Volatiles on Scavenging of Cucumber (Cucumis sativus L.) Reactive Oxygen Species
Source: Molecules. 2019 Sep 7;24(18):3263. doi: 10.3390/molecules24183263 (PMC6767350; doi:10.3390/molecules24183263)
Supplement: Supplementary file 1 [file molecules-24-03263-s001.zip › Supporting Information-Fan Yang/Table S2.pdf]

Table S2. Total 42 compounds of whole green garlic (in sealed desiccator)

Compound Name	**RT** (min)	**Area%**	Molecular Formula	Molecular Weight
2-Propenylidene cyclobutene	4.11	0.75	C7H8	92
Hexamethyl cyclotrisiloxane	5.17	6.82	C6H18O3Si3	222
Butyl cyclooctane	5.74	0.71	C12H24	168
2,3-Dimethyl-heptane	6.19	1.88	C9H20	128
1,1-bis(dodecyloxy)-Hexadecane	6.61	1.39	C15H19Cl2N3S	343
Nonane	7.40	0.70	C9H20	128
Cis-1-Ethyl-3-methyl-cyclohexane	7.59	0.70	C9H18	126
Propyl cyclohexane	8.22	0.72	C9H18	126
3,6-Dimethyl-undecane	8.30	3.02	C13H28	184
3-Ethyl-2-methyl-heptane	8.45	8.78	C10H22	142
1,1,2,3-Tetramethyl-cyclohexane	8.98	0.75	C10H20	140
1-Methyl-3-(2-methylpropyl)-cyclopentane	9.39	0.98	C10H20	140
Octamethyl cyclotetrasiloxane	9.67	4.80	C9H28O3Si4	296
1-Methyl-3-(2-methylpropyl)-cyclopentane	9.74	0.70	C10H20	140
Mesitylene	9.99	3.66	C9H12	120
Decyl vinyl ester carbonic acid	10.15	2.01	C13H24O3	228
4-Methyl-decane	10.72	3.44	C11H24	156
2-Cyclohexyl-Decane	10.80	1.63	C16H32	224
Eicosyl vinyl ester carbonic acid	11.15	1.12	C23H44O3	368
1-Ethyl-2,2,6-trimethyl cyclohexane	11.32	1.40	C11H22	154
1,2-Dimethyl-cyclooctene,	11.69	1.53	C10H18	138
Diallyl disulphide	12.27	15.30	C6H10S2	146
3-Tetradecyl ester trichloroacetic acid	12.40	2.10	C16H29Cl3O2	358
1-Nonylcycloheptane	12.54	0.72	C16H32	224
Phytol	12.80	2.85	C20H40O	296
Melezitose	12.89	1.11	C18H32O16	504
2-Methyl-trans-decalin	12.94	1.49	C11H20	152
2-Isopropylidene-5-methylhex-4-enal	13.17	1.76	C10H16O	152
Hexadecanethiol	13.46	0.89	C16H34S	258
1-Methyldecahydronaphthalene	13.57	1.21	C11H20	152
Decamethyl cyclopentasiloxane	13.68	2.46	C10H30O5Si5	370
Vinyl lauryl ether	14.86	3.89	C14H28O	212
2,6-Dimethyl-undecane	15.62	2.15	C13H28	184
2-Butyl-1,1,3-trimethyl-cyclohexane	15.83	4.00	C13H26	182
2,6,10-Trimethyl-tetradecane	17.02	1.27	C17H36	240
3,7,11-Trimethyl-1-dodecanol	17.14	1.55	C15H32O	228
Dodecamethyl cyclohexasiloxane	17.70	1.47	C12H36O6Si6	444
tert-Hexadecanethiol	19.38	0.79	C16H34S	258
2,6,10-Trimethyl tetradecane	25.95	1.40	C17H36	240
9-Hexyl-heptadecane	27.77	2.94	C23H48	324
2,6,10-Trimethyl-tetradecane	29.50	1.63	C17H36	240
Pentafluoropropionate octatriacontyl	31.15	1.54	C41H77F5O2	696
