# Supplementary figures and images for: Identification and Allelopathy of Green Garlic (Allium sativum L.) Volatiles on Scavenging of Cucumber (Cucumis sativus L.) Reactive Oxygen Species
Source: Molecules. 2019 Sep 7;24(18):3263. doi: 10.3390/molecules24183263 (PMC6767350; doi:10.3390/molecules24183263)

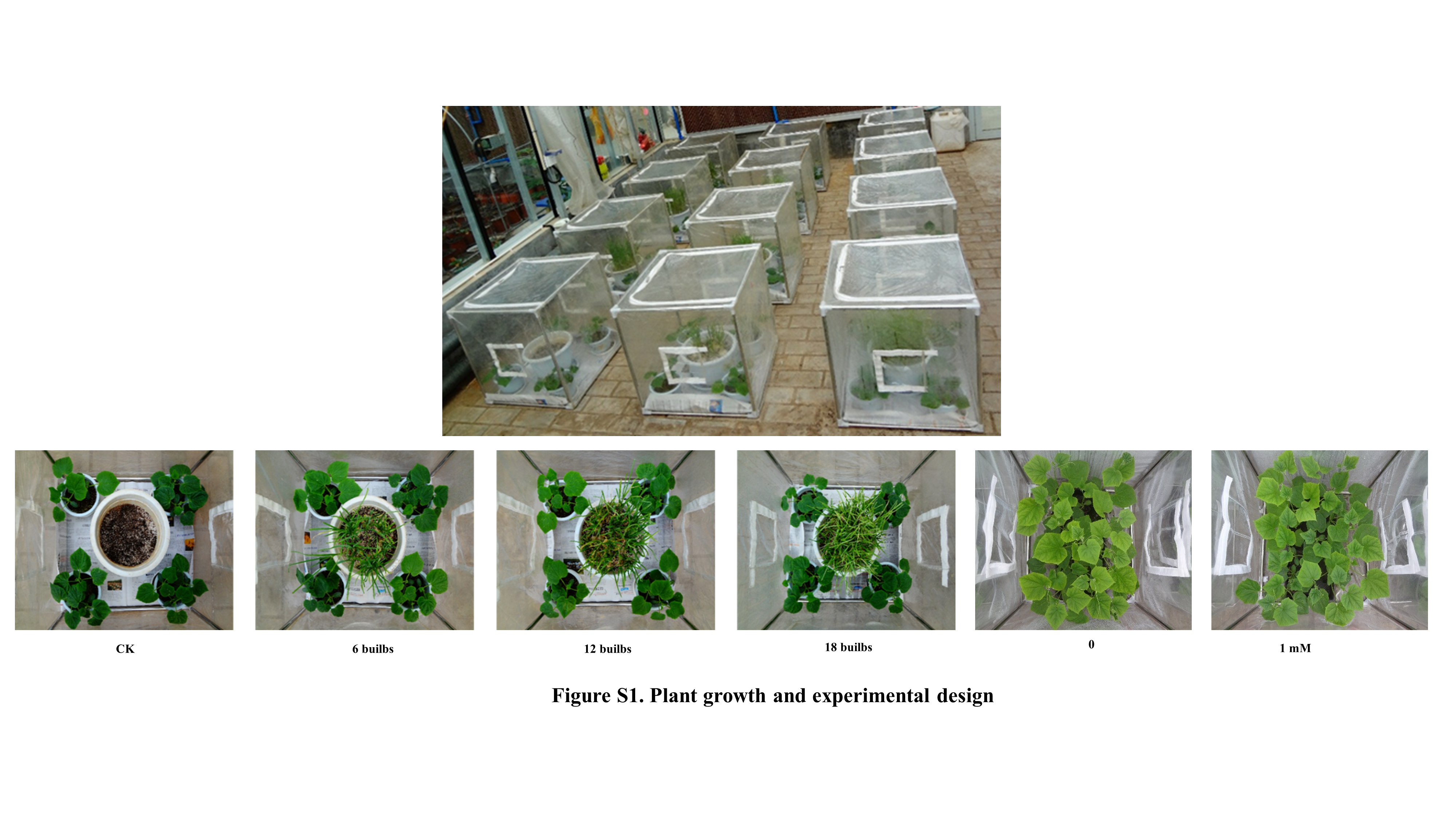

Supplement: Supplementary file 1 [file molecules-24-03263-s001.zip › Supporting Information-Fan Yang/Figure S1.PNG]

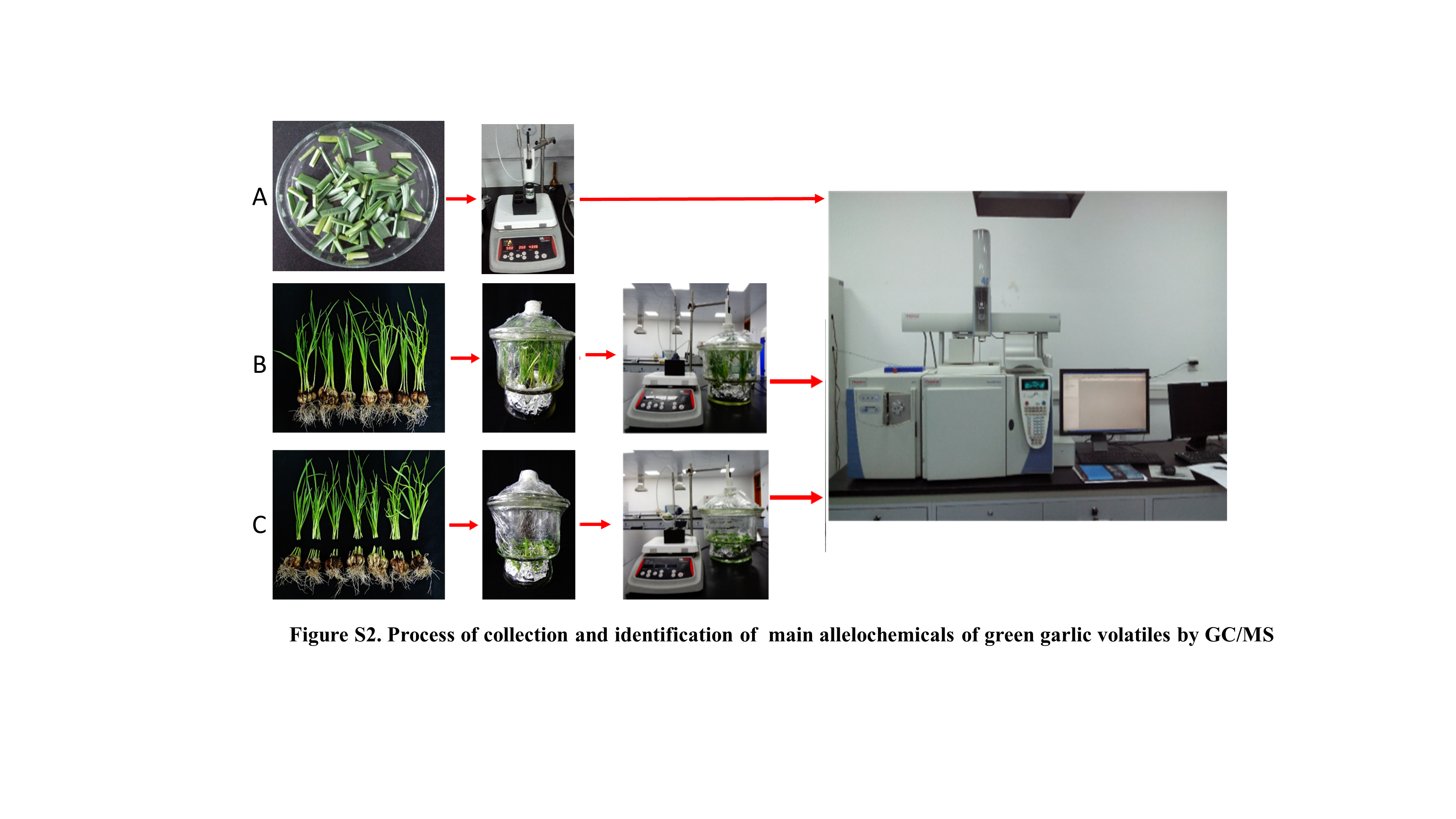

Supplement: Supplementary file 1 [file molecules-24-03263-s001.zip › Supporting Information-Fan Yang/Figure S2.PNG]

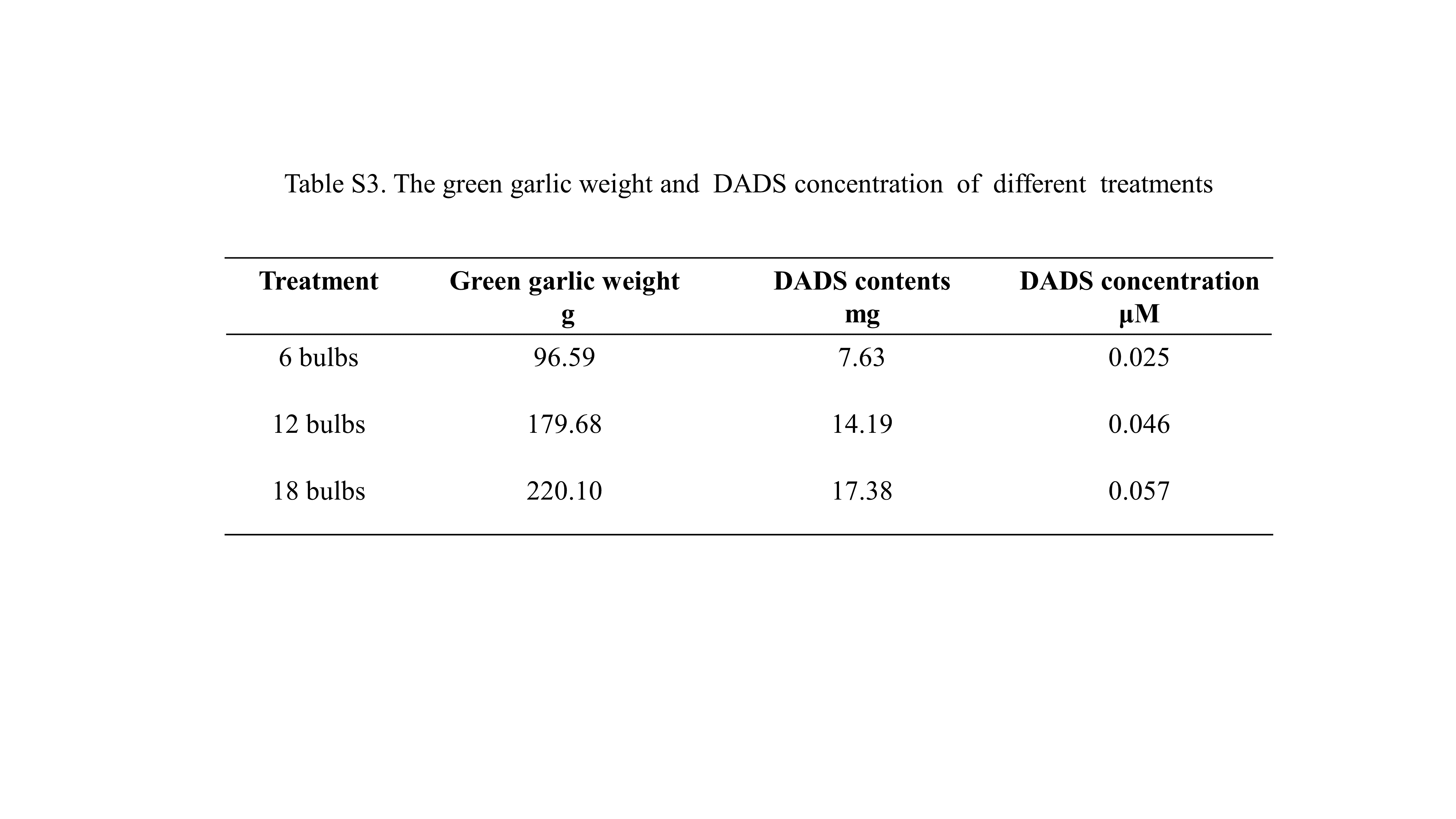

Supplement: Supplementary file 1 [file molecules-24-03263-s001.zip › Supporting Information-Fan Yang/Table S3.PNG]
